# Three-dimensional migration behavior of juvenile salmonids in reservoirs and near dams

**DOI:** 10.1038/s41598-018-19208-1

**Published:** 2018-01-17

**Authors:** Xinya Li, Zhiqun D. Deng, Tao Fu, Richard S. Brown, Jayson J. Martinez, Geoffrey A. McMichael, Bradly A. Trumbo, Martin L. Ahmann, Jon F. Renholds, John R. Skalski, Richard L. Townsend

**Affiliations:** 1 Pacific Northwest National Laboratory, P.O. Box 999, MSIN K9-33, Richland, WA 99332 USA; 2 Formerly at Pacific Northwest National Laboratory, Currently at Mainstem Fish Research, LLC, Richland, WA, 99354, USA; 3 U.S. Army Corps of Engineers, Walla Walla District, 201 N Third Ave, Walla Walla, WA 99362 USA; 4 School of Aquatic and Fishery Sciences, University of Washington, 1325 Fourth Avenue, Suite 1820, Seattle, WA 98101 USA

## Abstract

To acquire 3-D tracking data on juvenile salmonids, Juvenile Salmon Acoustic Telemetry System (JSATS) cabled hydrophone arrays were deployed in the forebays of two dams on the Snake River and at a mid-reach reservoir between the dams. The depth distributions of fish were estimated by statistical analyses performed on large 3-D tracking data sets from ~33,500 individual acoustic tagged yearling and subyearling Chinook salmon and juvenile steelhead at the two dams in 2012 and subyearling Chinook salmon at the two dams and the mid-reach reservoir in 2013. This research investigated the correlation between vertical migration behavior and passage routes. The depth distributions of fish within the forebays of the dams were significantly different from fish passing the mid-reach reservoir. Fish residing deeper in the forebay tended to pass the dam using deeper powerhouse routes. This difference in depth distributions indicated that the depth distribution of fish at the mid-reach reservoir was not related to behaviors of fish passing through certain routes of the adjacent dams. For fish that were detected deeper than 17.5 m in the forebays, the probability of powerhouse passage (i.e., turbine) increased significantly. Another important finding was the variation in depth distributions during dam passage associated with the diel period, especially the crepuscular periods.

## Introduction

Hydroelectric power production can result in mortality of turbine-passed fish due to several mechanisms^1^. At hydropower facilities, mortality of fish through different passage routes varies widely but is highest for passage through turbines^2^. To minimize the passage into hydropower turbines, it is important to understand the migration and passage behavior of fish through hydroelectric facilities^3,4^. Fish migration and passage behaviors have strong influence on the selection of routes at hydropower facilities^5–7^, and behaviors vary for different species. For example, silver eels prefer deeper routes rather than surface bypasses^8,9^ while juvenile salmonids are surface-orientated and thus surface spill is more effective for their downstream migration^10^. In the Pacific Northwest, intensive studies^11–13^ have been performed to monitor the behavior and survival of downstream-migrating juvenile salmonids for the purpose of increasing the survival rate at hydropower facilities where turbine passage leads to high mortality. Hydropower facilities leading to declines in migratory species’ populations is a common environmental concern for the international community^14–18^. For the purpose of guiding fish away from dangerous turbines effectively to increase the survival rate, technologies that offer safer passage (fishways, bypass facilities, etc.) are generally pursued^19–22^.

**Figure 1:**
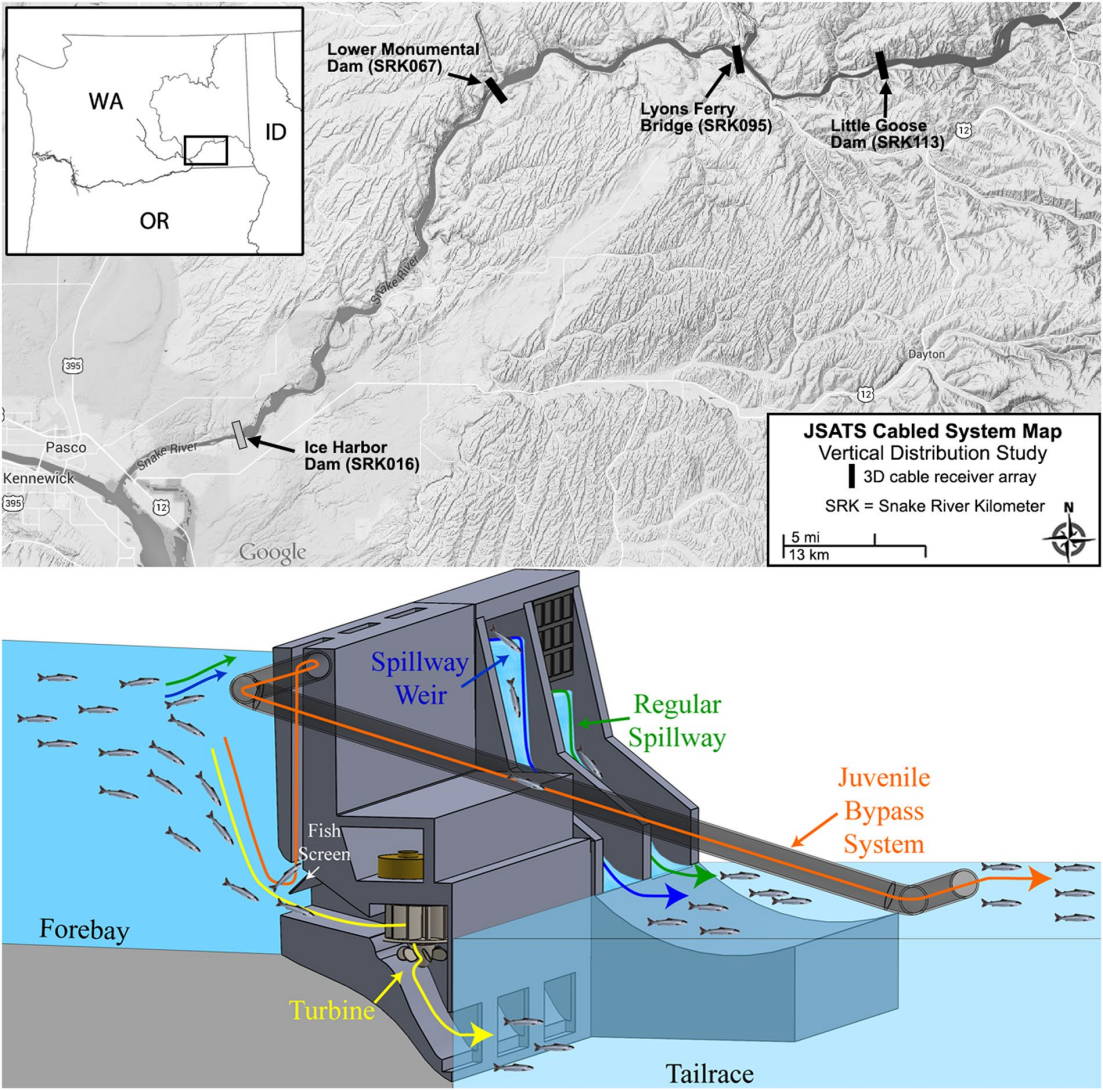
(Top) Locations of JSTAS-Cabled Hydrophone Arrays on the Snake River (Little Goode Dam, Lyons Ferry Bridge and Lower Monumental Dam) in Washington State, USA (the source of the map is Google Maps); (Bottom) A schematic diagram (created referring to NOAA Fisheries West Coast Region^44^) from of the four passages routes at Little Goode Dam and Lower Monumental Dam.

In order to reduce the mortality of juvenile salmonids attributed to turbine passage, many dams in the Columbia River Basin have four possible passage routes for downstream migrating fish: 1) spillway (Tainter gate, “deep” spill), 2) temporary or removable spillway weir (SW; “surface” spill), 3) powerhouse turbines, and 4) juvenile bypass system (JBS). These four passage routes are installed at different depths below the water surface with turbine passage being the deepest route, as shown in the schematic diagram of the four passage routes (Fig. 1). The four passage routes have two categories, traditional structures (regular spillway and turbines) and those specifically added to improve fish passage (surface spill and JBS). Although still not well understood, the understanding of route selection behavior is important since these routes differ in their mortality effects on fish and thus route selection behavior is important information for mitigating the impacts of dams on fish populations^23,24^. Fish search in the forebay for a possible route when they are approaching the dam. The acclimation depth (the depth at which fish achieve neutral buoyancy) of fish prior to dam passage may determine which route is the most accessible method of passage. Thus it is critical to understand the vertical migratory behavior of fish before they approach hydroelectric dams. Vertical migration is a typical phenomenon for many fish species, and diel changes associated with light intensity are the main proximate trigger^25–27^. Hence, it is essential to investigate the behavior of different fish species during dam passage that may be associated with these diel changes.

The research presented in this study is closely related to two previous studies^28,29^. One of the related studies^28^ provided methodology details of how acclimation depth was estimated and evaluated for individual fish at Little Goose Dam and Lower Monumental Dam (Fig. 1) prior to passage. It focused only on the depth distribution of juvenile salmonids in the forebays prior to passage through turbines or the JBSs. The statistical analyses developed^28^ were also used to conduct pairwise comparisons of the depth distributions between sample groups in this research. Another previous study^29^ described how a cabled acoustic telemetry system was designed and installed at the mid-reach reservoir (Lyons Ferry Bridge) to collect high-resolution 3D tracking data. It provided the background of hardware and methods for data collection involved in this study. This study contributes to the further understanding of the continuous depth distribution of juvenile salmonids on their seaward migration through the two dams and the mid-reach reservoir. We used a large set of 3-D fish track data from approximately 33,500 tagged juvenile salmonids (*Oncorhynchus tshawytscha* and *Oncorhynchus mykiss*) during the migration seasons of 2012 and 2013. Such a large data size helped to ensure that the sample size (related to precision) used in the statistical analyses was adequate when the sample groups in comparisons were classified according to species, year, location(dam or mid-reach reservoir), and diel period (day or night).

The focus of this study is the correlation between vertical migration behavior (depth distribution) and passage route. To investigate the dependence of passage routes on depth distribution, the objectives of this study are answers to the following questions: 1) Are fish residing deeper in the forebay while approaching the dam more likely to pass through deeper routes? 2) Is the depth distribution of fish at a mid-reach reservoir related to the passage route depth at upstream or downstream dams? 3) What is the influence of diel period on depth distribution? 4) What is the probability of a fish detected at a specific location in the forebay and ultimately passing through a certain passage route? In this study we focus on presenting new results, as opposed to presenting new methods^28,29^. The continued depth distributions at forebays and at a mid-reach location were statistically described. We also provided the depth distribution comparisons of all four passage groups: spillway-weir-passed fish, regular-spillway-passed fish, JBS-passed fish and turbine-passed fish. The spatial distribution of passage probability by four routes was calculated and illustrated. The temporal distribution was represented by 24 h profiles to reveal more information related to the influence from diel period.

## Results

### Mid-reach *vs*. Forebay

The subyearling Chinook salmon at the mid-reach reservoir tended to move at shallower depths in the water column than in the forebays at both LGS and LMN in 2013. The median acclimation depth at LFB was 1.2 m shallower than at LMN and 2.0 m shallower than at LGS. At LGS, 95% of the fish resided at depths shallower than 15.6 m in the water column, at LMN they were shallower than 15.4 m, and at LFB they were shallower than 12.3 m. All the depth distribution comparisons of sample groups included in this category were significantly different (P < 0.001; Table 1).

**Table 1:** Median depth at which all subyearling Chinook salmon were detected within the forebays of LGS and LMN and at LFB during 2013.

Location A	Location B	Location A			Location B		
		Sample Size	Most Common Depth (95%) (m)	Median Depth (m)	Sample Size	Most Common Depth (95%) (m)	Median Depth (m)
LGS	LMN	2428	**15.6** (15.2, 16.8)	**7.0** (6.7, 7.3)	5388	**15.4** (15.2, 16.0)	**6.2** (6.1, 6.5)
LGS	LFB	2428	**15.6** (15.2, 16.8)	**7.0** (6.7, 7.3)	4224	**12.3** (12.0, 12.7)	**5.0** (4.7, 5.1)
LFB	LMN	4224	**12.3** (12.0, 12.7)	**5.0** (4.7, 5.1)	5388	**15.4** (15.2, 16.0)	**6.2** (6.1, 6.5)

95% CI are listed in parentheses. The depth at which 95% of the fish were found at or were shallower than is also shown. All of the comparisons of depth distribution between two locations were significantly different (P < 0.001).

Although the forebay acclimation depth distributions were significantly different from each other (P < 0.001) at LMN and LGS for fish from the 2013 study (Table 1), the depth distributions were similar, with the difference between the most common depths being only 0.2 m.

### Mid-reach Depth Distribution versus Passage Depth

Comparisons of depth distributions at LFB are shown in Table S1 in the Supplementary Information. None of the comparisons were significantly different (P ≥ 0.05). Regardless of the routes fish passed at the dams, their depth distributions were similar at the mid-reach reservoir. The median acclimation depth at LFB was in a relatively narrow range of 5.0 to 6.0 m. At LFB, most (95%) of the fish were found in the water column at depths above 11.4 to 13.2 m (46% to 53% of total river depth).

### Dependence of Passage Routes on Depth Distribution

The depth distribution comparisons between spillway-weir-passed fish and regular-spillway-passed fish were significantly different (P < 0.001; Table S2 in the Supplementary Information). Most spillway-weir-passed fish resided in the top 13.6 m of the water column while most regular-spillway-passed fish swam in the top 17.9 m.

**Figure 2:**
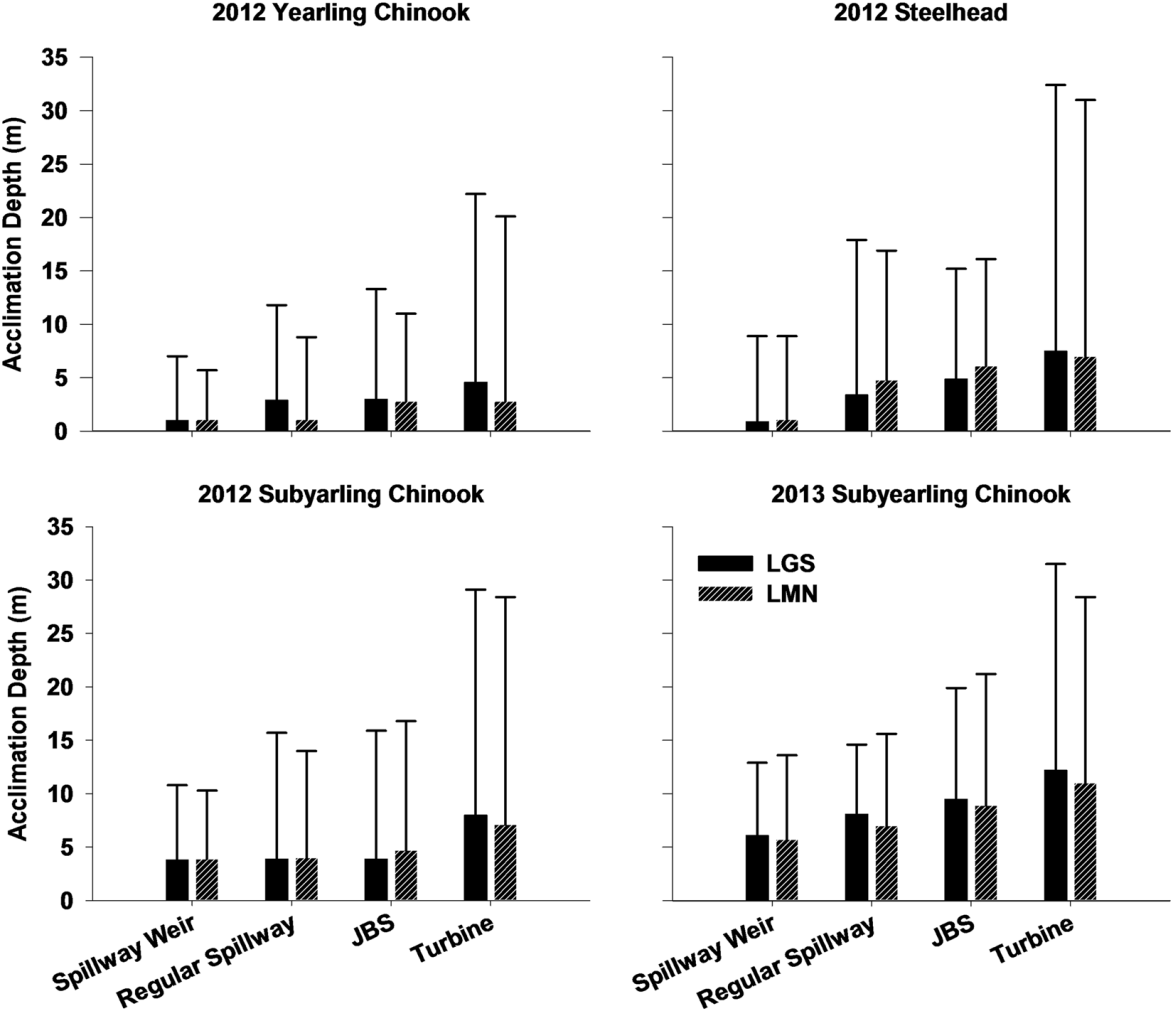
Median acclimation depth (edge of solid bar) and most common (95%) acclimation depth (edge of whisker) of acoustic tagged juvenile fish that passed through the four different routes at LGS and LMN in 2012 and 2013.

All comparisons between JBS-passed fish and regular-spillway-passed fish were significantly different (Table S3 in the Supplementary Information). Fish that passed through the regular spillway were detected shallower in the forebay than JBS-passed fish. Across species, years, and locations, juvenile salmonids that passed through deeper routes swam deeper in the water column when approaching the dam (Fig. 2). This was especially evident for turbine-passed fish, which were detected at much deeper depths (≤32.4 m).

**Figure 3:**
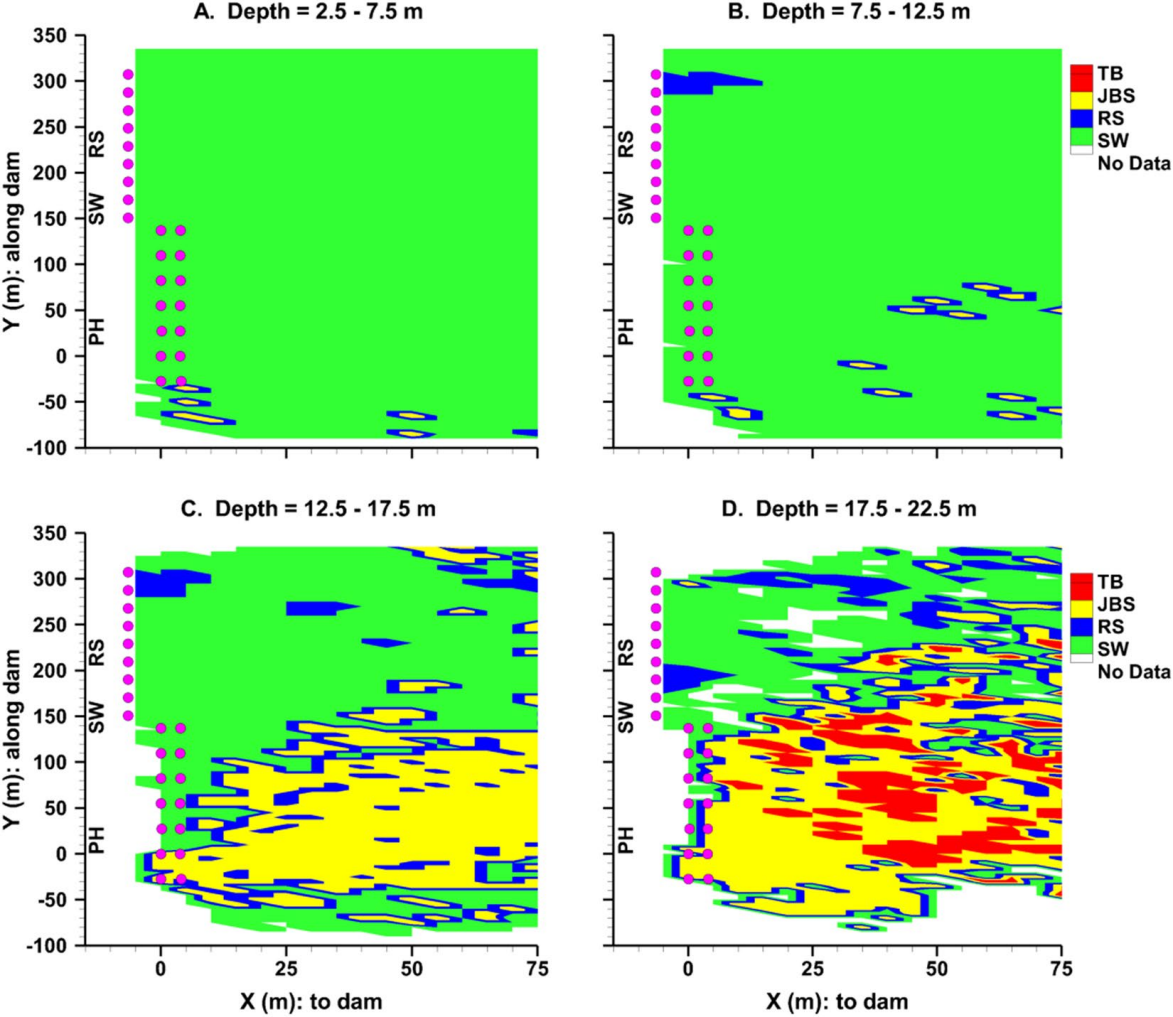
Distribution of highest passage probability for subyearling Chinook passed by four different routes (“TB” = turbine; “JBS” = juvenile bypass system; “RS” = regular spillway; “SW” = spillway weir) at LGS during 2013 summer at four different depth levels. (**A**) Depth = 2.5 to 7.5 m; (**B**) Depth = 7.5 to 12.5 m; (**C**) Depth = 12.5 to 17.5 m; (**D**) Depth = 17.5 to 22.5 m. Solid purple dots are locations of hydrophones at piers showing the location of the dam face. “PH” indicates the powerhouse section. The highest probability among passage routes was represented by the corresponding color.

The trends of depth distribution before dam passage in the LGS forebay in 2013 were reflected by the passage probability contour maps in a plane view (Fig. 3). For subyearling Chinook salmon that were detected at least once shallower than 12.5 m (Fig. 3A and B), the fish were more likely to be guided by the spillway weir across the forebay area than the other routes, except for a small area deeper than 7.5 m near the north end of spillway. For subyearling Chinook that were at least once detected below 12.5 m in front of the powerhouse (Fig. 3C), the probability of passing the dam through the JBS increased to its highest (more than 50% for yellow area in Fig. 3C). For the spillway section of the forebay, the probability of passing the dam through the spillway weir (SW) was still high when fish were detected near any part of the spillway. However, for areas farther away from the spillway section, the probability that other routes would be used increased. For fish that were detected deeper than 17.5 m (Fig. 3D), the probability of powerhouse passage (i.e., through either the JBS or turbine) increased significantly and covered a larger forebay area while the probability of spill weir passage continued to decrease.

### Influence of Diel Period

During 2013, subyearling Chinook salmon at the mid-reach reservoir (LFB) tended to be 2.8 m shallower in the water column during the day than during the night (Table 2). For all fish that passed LGS and LMN, the median acclimation depths were 2.7 m and 1.9 m deeper during the day than at LFB, respectively. The median acclimation depths were 0.9 m and 2.4 m shallower during the night at LGS and LMN than at LFB, respectively. For all night-passed fish, most (95%) were detected in the top 26 m of the water column in the forebays of LGS and LMN (~74% of local river depth), while these fish only resided in the top 15 m of the water column at LFB (~60% of local river depth).

**Table 2:** Median depth during day and night at which subyearling Chinook salmon from 2013 were detected across all sites in the Snake River (LFB, LGS, and LMN).

Sample Group A				Sample Group B			
	Sample Size	Most Common Depth (95%) (m)	Median Depth (m)		Sample Size	Most Common Depth (95%) (m)	Median Depth (m)
Day passed at LFB	3042	**10.7** (10.4, 11.0)	**4.4** (4.3, 4.6)	Night passed at LFB	1181	**15.0** (14.6, 15.6)	**7.2** (6.7, 7.4)
Day passed at LFB	3042	**10.7** (10.4, 11.0)	**4.4** (4.3, 4.6)	Day passed at LGS	1920	**13.4** (13.2, 14.0)	**7.1** (6.8, 7.3)
Day passed at LFB	3042	**10.7** (10.4, 11.0)	**4.4** (4.3, 4.6)	Day passed at LMN	4676	**14.0** (13.8, 14.3)	**6.3** (6.2, 6.5)
Night passed at LFB	1181	**15.0** (14.6, 15.6)	**7.2** (6.7, 7.5)	Night passed at LGS	508	**25.9** (23.5, 28.3)	**6.3** (3.2, 9.1)
Night passed at LFB	1181	**15.0** (14.6, 15.6)	**7.2** (6.7, 7.5)	Night passed at LMN	711	**24.1** (21.9, 26.6)	**4.8** (3.7, 7.0)

95% CI are listed in parentheses. The depth at which 95% of the fish were found at or were shallower than was is shown. All of the comparisons of depth distribution between two sample groups were significantly different (P < 0.001).

**Figure 4:**
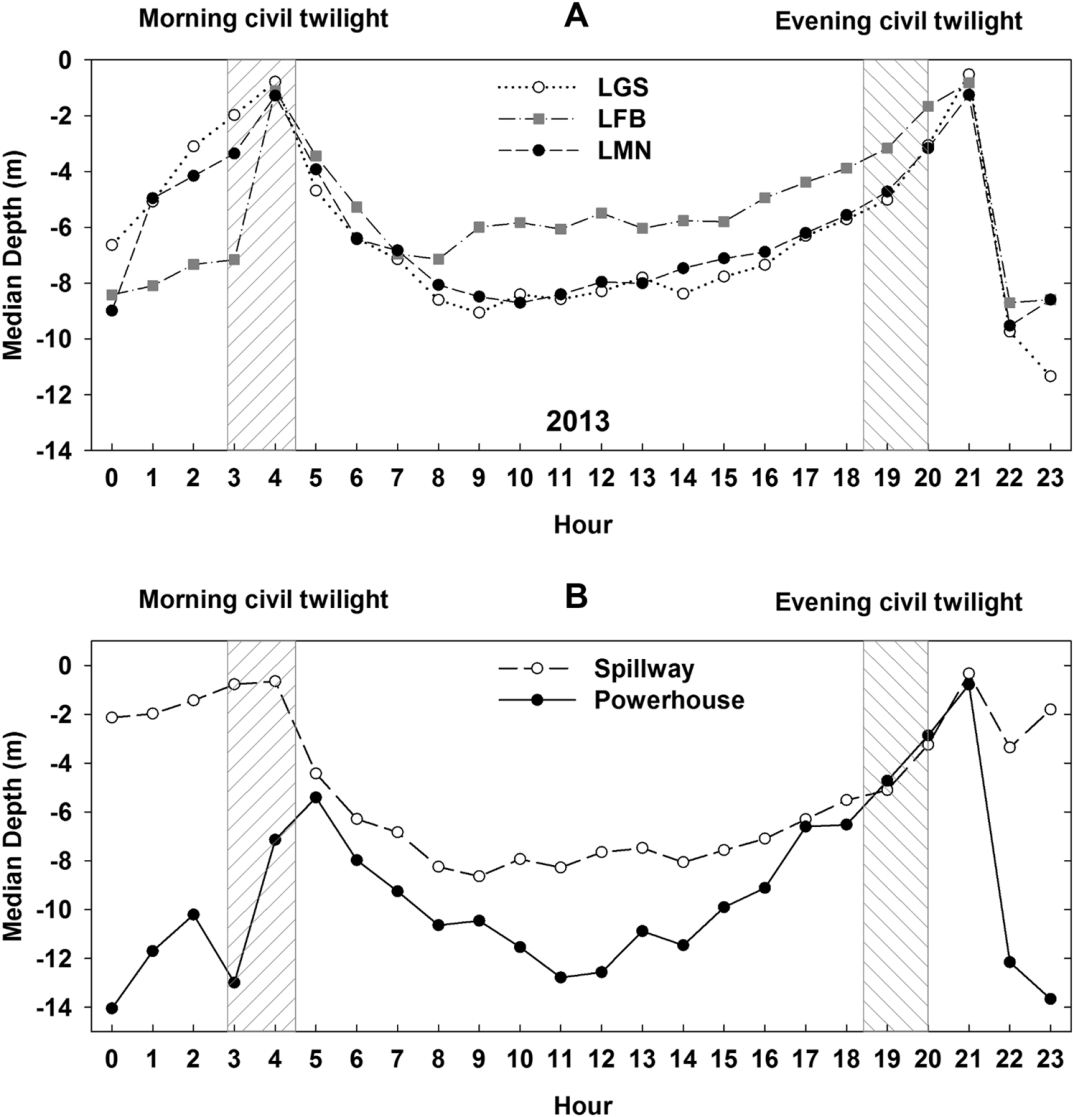
Median depth of acoustic tagged subyearling Chinook salmon. (**A**) Fish were detected at LGS, LFB, and LMN in 2013 in each hour of the day; (**B**) Fish passed spillway and powerhouse in each hour of the day that juvenile fish were detected within 75 m of the dam face in the forebays of LGS during 2013. The dashed areas represent the two periods of civil twilight varying from April to August, respectively.

Subyearling Chinook salmon were observed to be shallowest around the dusk and dawn periods at all of the study sites (Fig. 4A). The pattern of shallowest depths during the crepuscular periods was also observed when the tagged fish were divided by passage route (Fig. 4B). Similarly at LFB subyearling Chinook salmon resided shallower during the day than during the night. Within 75 m of the dam face in the LGS forebay during 2013, subyearling Chinook salmon that passed through the spillway were much deeper during the day than during the night (Fig. 4B), while powerhouse-passed subyearling Chinook salmon were at relatively similar depths during day and night hours except during crepuscular periods. Since a much higher number of salmonids passed the dam through the spillway compared to the powerhouse, when all passage routes were combined at LGS and LMN in 2013 (Fig. 4A) subyearling Chinook salmon were on average deeper during the day than the night at both locations. This trend was the opposite of the pattern observed at LFB.

**Figure 5:**
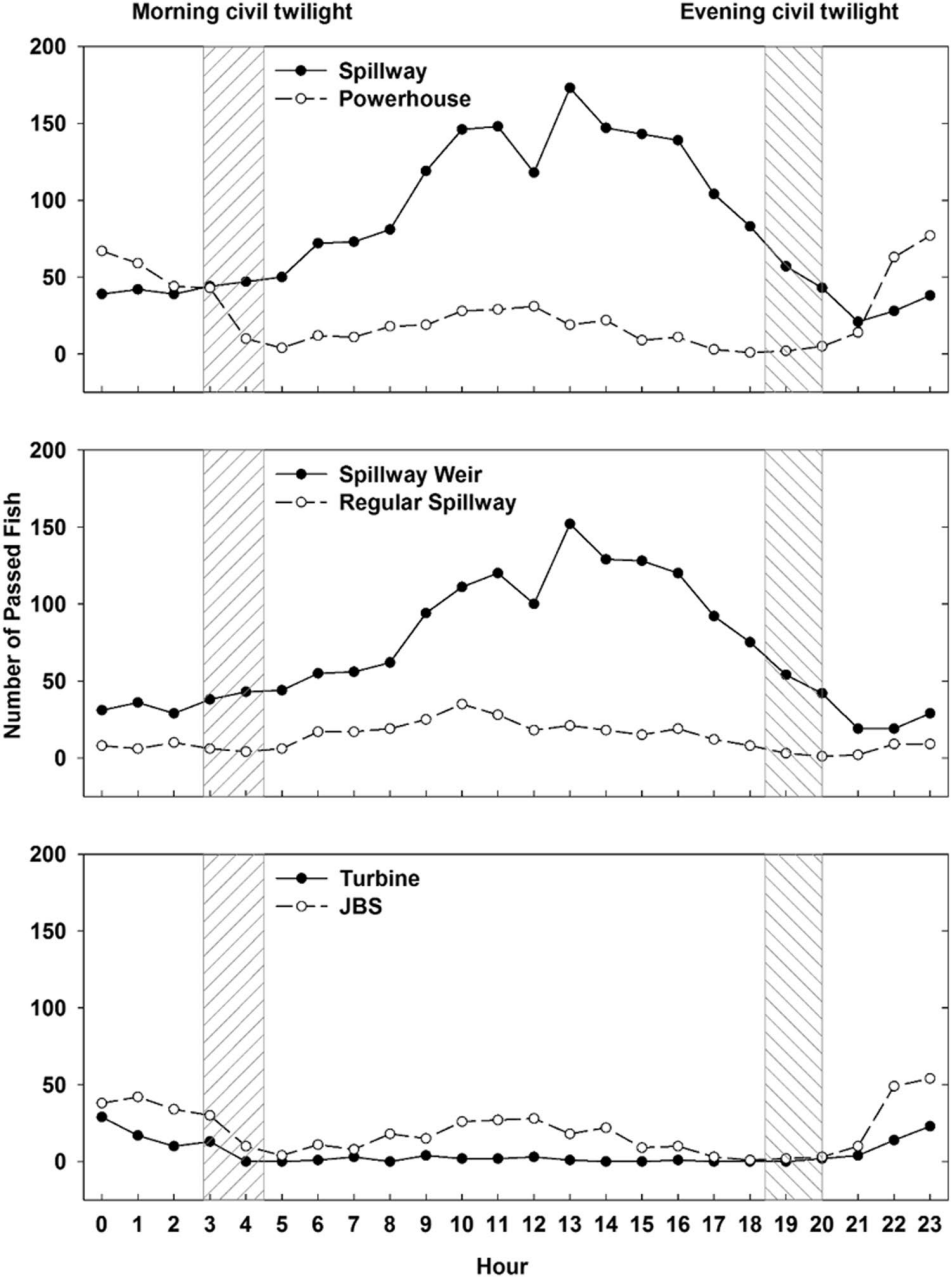
Number of acoustic tagged subyearling Chinook salmon that passed LGS in 2013 for each hour of the day. (Top) Comparison between spillway and powerhouse passage; (Middle) Comparison between spillway weir and regular spillway passage; (Bottom) Comparison between turbine and JBS passage. The dashed areas represents the two periods of civil twilight varying from April to August, respectively.

For the 2,595 detected salmonids that passed LGS in 2013 (Table S4 in the Supplementary Information**)**, the peak of dam passage through the surface weir happened around noon (Fig. 5). During the day 72% of the total salmonids passed the dam while only 28% passed during the night. For the 1,678 fish that passed the dam through the surface weir, most (83%) of them passed during the day while only a small percentage (17%) passed that route during the night. The pattern was similar for fish that passed through the regular spillway. Overall, approximately half (54%) of all dam passed fish traveled through the surface weir during the day. The patterns were different for powerhouse-passed fish; 36% of those fish passed the dam during the day, while 64% passed during the night. For fish that traveled through the JBS, 42% passed during the day and 58% passed during the night. The difference in percentage between day and night for fish that passed the JBS was not significant. However, for fish that passed through the turbine, 112 out of 129 fish passed during the night. Few fish passed the dam through the turbines during the day (only 17 passed through a turbine during the day, 0.6% of all passed fish).

**Figure 6:**
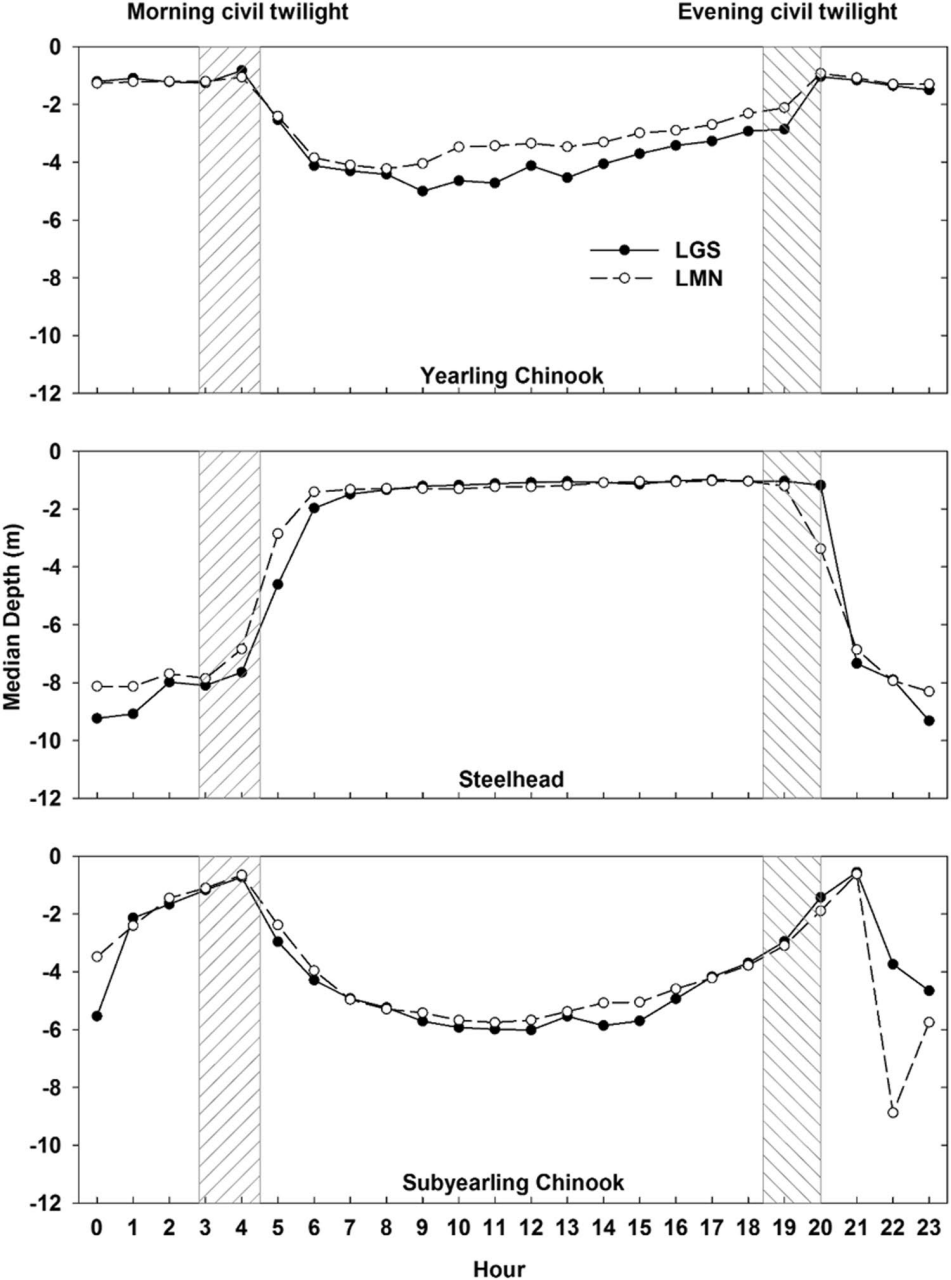
Median depth of acoustic tagged yearling Chinook salmon (Top), steelhead (Middle) and subyearling Chinook salmon (Bottom) in each hour of the day that juvenile fish were detected within 75 m of the dam face in the forebays of LGS and LMN during 2012. The dashed areas represents the two periods of civil twilight varying from April to August, respectively.

The depth distribution based on diel period varied among the different species/life history types (Fig. 6). For yearling Chinook salmon and subyearling Chinook salmon, when considering all passage routes combined at both locations in 2012, differences in depths between these two life history types were more apparent during the day and less apparent during the night, especially during the crepuscular periods. Yearling Chinook salmon resided very shallow (not deeper than 2 m) in the water column during the night. Depth distributions of juvenile steelhead were very different than juvenile Chinook salmon. Juvenile steelhead were much shallower during the day than during the night. The median depths were less than 2 m at LGS and LMN in 2012 during the day. Differences among species can be quantified through day vs. night comparisons (Table 3). The median depth of juvenile steelhead that passed through the spillway was 5.7 m and 4.8 m deeper during the night at LGS and LMN in 2012 respectively. For fish that passed through the spillway during the night, the median depths of yearling Chinook salmon were the same as subyearling Chinook salmon at LGS and LMN in 2012. However, subyearling Chinook salmon occupied deeper depth levels during the day.

**Table 3:** Median depth during day and night at which three types of juvenile salmonids passed spillway routes were detected within the forebay of LGS and LMN during 2012 and 2013.

Year	Species	Location	Passed during day			Passed during night		
			Sample Size	Most Common Depth (95%) (m)	Median Depth (m)	Sample Size	Most Common Depth (95%) (m)	Median Depth (m)
2012	CH1	LGS	767	**7.9** (7.5, 8.9)	**2.3** (1.8, 2.9)	280	**10.7** (8.4, 12.9)	**0.9** (0.7, 1.0)
2012	ST	LGS	715	**8.4** (7.1, 11.5)	**0.9** (0.9, 1.0)	202	**17.1** (14.4, 20.2)	**6.6** (5.5, 7.5)
2012	CH0	LGS	1535	**10.2** (10.0, 10.8)	**4.1** (3.8, 4.3)	330	**19.7** (17.8, 22.9)	**0.9** (0.7, 1.5)
2012	CH1	LMN	2204	**6.1** (5.9, 6.5)	**1.2** (1.2, 1.3)	852	**7.5** (6.4, 8.6)	**1.0** (1.0, 1.1)
2012	ST	LMN	1951	**7.2** (6.7, 8.0)	**1.1** (1.1, 1.2)	651	**15.9** (13.4, 19.3)	**5.9** (5.4, 6.4)
2012	CH0	LMN	4317	**9.8** (9.6, 10.2)	**4.1** (4.1, 4.3)	806	**18.6** (17.2, 20.7)	**1.0** (0.9, 1.2)
2013	CH0	LGS	1619	**12.9** (12.6, 13.1)	**6.8** (6.4, 7.0)	197	**16.2** (14.5, 20.7)	**0.9** (0.5, 1.8)
2013	CH0	LMN	4253	**13.4** (13.3, 13.8)	**6.2** (6.1, 6.4)	492	**18.9** (17.0, 20.5)	**2.7** (2.3, 3.5)

95% CI are listed in parentheses. The depth at which 95% of the fish were found at or were shallower than is also shown. All of the comparisons of depth distribution between day-passed and night-passed were significantly different (P < 0.001). CH0 = subyearling Chinook salmon; CH1 = yearling Chinook salmon; ST = steelhead.

## Conclusion

In our study, high resolution 3D tracking data contributed to the understanding of detailed vertical migration behavior, the distribution profiles of acclimation depth of juvenile fish, on their continuous downstream migration passing one dam, a mid-reach reservoir and another dam. The acclimation depth is positively correlated to the depth of passage routes. Juvenile salmonids that passed through deeper routes swam deeper in the water column when approaching the dam. For fish that were detected deeper than 17.5 m, the probability of powerhouse passage (i.e., turbine) increased significantly. The depth distribution of fish was not influenced by their previous migration or passage behavior. Diel period has a strong influence on the vertical migration behavior and varied among the different species/life history types. For subyearling Chinook salmon, they were observed to be shallowest during crepuscular periods at dams, and surface weir passage during the day was the most favored passage route. Most of the turbine passage happened during the night.

## Discussion

The research indicated that subyearling Chinook salmon tended to be deeper in the water column as they traveled through the forebay of dams on the Snake River (LGS and LMN) compared to the mid-reach reservoir at LFB. The analysis showed that most (95%) of the subyearling Chinook salmon migrating through the forebay of LGS and LMN were detected at depths shallower than 18 m; whereas, most were detected shallower than 13 m at the mid-reach reservoir. The bathymetry of the Snake River may have contributed to these observations, since in general, the mid-reach section of the river is shallower (up to 25 m deep) than in the forebays of the dams (up to approximately 35 m deep). Thus, when expressed as the percentage of total local river depth, depths in the forebay (18 m/35 m = 51%) and at mid-reservoir (13 m/25 m = 52%) were similar. Other potential factors also may have contributed to the differences in the detection depth between fish in the forebays of dams and the mid-reach reservoir, including wind levels^30^, water temperature^31^, substrate^32^, turbidity and the depth where predators^33^ were present.

The acclimation depths identified in this analysis are similar, but not identical, to those estimated in the laboratory research of Pflugrath *et al*.^34^, who reported that the average maximum depth at which juvenile Chinook salmon could maintain neutral buoyancy was 6.7 m (range 4.6 to 11.6 m). The results of Pflugrath *et al*.^34^ are more similar to the depths of fish that were tracked at LFB (median 5.0 m) than within the forebays of dams where the medians were 6.2 to 7.0 m. This may indicate that fish were searching more of the water column when in the forebays. Fish at LFB may have been acting more naturally and swimming predominately in a way that would conserve energy (i.e., it is more likely they were detected at their acclimation depths). For a fish, swimming at the depth at which it is neutrally buoyant is an important strategy for conserving energy^35^, which can be of particular concern during the large seaward migrations of juvenile salmonids. By comparison, fish that are searching a larger portion of the water column to attain passage within the forebays could possibly be negatively buoyant while at greater depths.

Another topic of interest was if fish swam deeper at upstream (mid-reach or another dam) will go through deeper routes and vice versa. The analysis performed in this study did not find any preference for selection of passage routes at a dam relative to upstream depth distributions. For example, turbine-passed fish were not found to reside deeper at the mid-reach location or pass through deeper routes at an upstream dam. The passage behavior of the juvenile salmonids tagged in this study were more likely determined by their behavior through searching and diving^28^. However, it was also revealed that fish generally swam at relatively shallower depths at the mid-reach reservoir where there is little influence from a nearby hydroelectric facility. At this location, fish were predominately detected at depths less than 13 m in the water column. Hence when first approaching a dam, most fish were more easily guided to safer passage routes (i.e., through the spillway weir, regular spillway, and JBS) rather than through the turbines (deeper than 18 m). Thus, the information of depth distribution at the mid-reach location was helpful to understand why a larger percentage of fish passed through shallow routes. Our analysis indicated that for fish that did pass through the turbines, which accounted for only 5% of all passed fish, they were found to reside significantly deeper in the forebay before reaching the near-dam flow zone, than fish that passed through other routes. The spillway weirs at dams, which provide downstream passage via surface spill, were generally installed in the middle of the structure between the regular spillway and the powerhouse. Test on these weirs were performed^36^ by US Army Corps of Engineering. The spillway weirs were the preferred passage route for fish that were shallow in the water column (≤10 m) and the first encountered passage route when fish searched along the dam face of the powerhouse towards the spillway (Fig. 3). Before reaching the dam face, most fish that swam deeper (≤17.5 m) in the forebay in front of the powerhouse passed through the turbine or JBS.

Another important finding in this study was the difference in dam passage associated with the diel period. Subyearling Chinook salmon were mostly located near the water surface around dusk and dawn. This phenomenon may vary among different species life history types^37^, and could support the theories of Clark and Levy^38^ who stated that the ratio of mortality risk in relation to feeding rates for juvenile salmon reaches a minimum at intermediate levels of light intensity (intervals at dawn and dusk). Juvenile salmonids may optimize the tradeoff between food intake and predation risk by migrating into the near-surface waters to feed within the two crepuscular time windows. By comparing the depth distributions of day-passed and night-passed fish at LFB, subyearling Chinook salmon were detected deeper in the water column during the night and were detected at shallower depths during the day. It is possible that this is one of the major reasons why most of the day-passed fish passed through spillway routes and most night-passed fish passed through the powerhouse routes. The higher potential for passing via powerhouse routes at deeper depths may have been the easiest passage for subyearling Chinook salmon during the night, since they tended to be detected deeper in the water column at night. Li *et al*.^28^ also found that fish took a much longer time to pass dams through powerhouses during the day than during the night. Li *et al*.^28^ revealed that the influence of the diel period with respect to fish behavior depends on the species or life history type. In this study, for species in the 2012 comparison (Fig. 6), yearling Chinook salmon were found to be the least influenced by the diel period and they exhibited the least depth change between day and night during their presence in the forebay.

For dam operation management, it is helpful to understand how different fish species occupy different depths in the water column of forebays during day and night. Findings of this study can be used to guide the dam operation to mitigate fish mortality due to the turbine passage. As shown in the study, turbine-passed fish reside significantly deeper in the forebay before reaching the near-dam flow zone than fish passing through other routes. Dam operators could use the distribution of residential depths of turbine-passed fish to justify the lowering of JBS guidance screens to lure more fish through JBS passage or to design and implement alternate fish passages to guide deep residence fish. The diel effects of subyearling Chinook salmon: fish tend to stay deeper in the water column during the night, at shallower depths during the day, and near the water surface around dusk and dawn, could be used to help spillway and RSW operations to guide fish through these two passage routes. For example, since fish were deeper at night, when most turbine passage occurred, discharge through turbines could be decreased and balanced by increased discharge through deep spill.

## Methods

### Study Sites

The study was performed at two dams (Little Goose Dam and Lower Monumental Dam) and one mid-reach reservoir (Lyons Ferry Bridge on Snake River; Fig. 1). Little Goose Dam (LGS) is located on the Snake River, 113 river kilometers (rkm) upstream from the confluence with the main stem of the Columbia River, in southeast Washington State. The dam consists of a six-unit powerhouse, an eight-bay spillway, and a temporary surface weir. Lower Monumental Dam (LMN), similar in design to LGS, is located 46 rkm downstream of LGS. Fish implanted with JSATS acoustic transmitters were detected using JSATS-cabled hydrophone array^28^ deployed on the dam faces of LGS and LMN^39^ in 2012 and 2013. As described in Fig. 1, four routes exist at LGS and LMN. The spillway weir (surface spill) is installed no more than 6 m below the water surface. A regular spillway bay (deep spill) is no more than 15 m deep. The top of the turbine intake is about 19 m below the water surface at both dams. A JBS screen was installed above the turbine intake, for the purpose of guiding fish up through channels constructed in the dam (indicated by orange line in Fig. 1), routing them away from turbines and carrying them to the downstream side of the dam. The depths of river beds relative to the water surface (termed total river depth) at LGS and LMN are both approximately 35 m.

The Lyons Ferry Bridge (LFB; Washington State Highway 261) study site is located in a mid-reach reservoir between LGS and LMN, 18 rkm downstream of LGS and 28 rkm upstream of LMN. Near the LFB mid-reach study site, the Palouse River enters the Snake River from the north approximately 0.5 km upstream, and the Tucannon River enters from the south approximately 6 km upstream of the study site. In 2013, LFB provided a rigid structure to attach JSATS-cabled hydrophone systems^29^. The water depth at this location ranges from 12 m (near shore) to 25 m (total river depth at LFB), which represent the typical mid-reach depths of this area.

### 3-D Tracking Data Collection

Acoustic telemetry is considered one of the most effective approaches for monitoring fish behavior^40^. The Juvenile Salmon Acoustic Telemetry System (JSATS) is a well-developed tool that has been used extensively for over 10 years to monitor the behavior and survival of juvenile salmonids migrating through rivers, reservoirs, and hydroelectric dams on their way to the Pacific Ocean^41–43^. The deployment of the JSATS-cabled hydrophone (Model 2010, Sonic Concepts Inc., Seattle, WA, USA) arrays on the dam face of LGS and LMN facing forebay was described in Li *et al*.^28^. For each pier, two hydrophones were deployed at two depths at the powerhouse and spillway. All shallow hydrophones were about 3.5 m below the water surface and the deep hydrophones were 25 m deeper at the powerhouse and 8–9 m deeper at the spillway than shallow hydrophones. At LFB^29^, the design of the hydrophone array was similar along the bridge piers with the vertical spacing between the shallow and deep hydrophones being approximately 9.3 to 15.7 m. The horizontal spacing is much larger than the dam hydrophones due to the larger spacing of the piers. The JSATS acoustic tags used in studies in conducted in 2012 and 2013^24^ were manufactured by ATS (Advanced Telemetry Systems). Tags (model number SS300) were 10.79 mm long, 5.26 mm wide, 3.65 mm high, and weighed 0.346 g in air with a tag volume of 0.125 mL. The tags had a nominal transmission rate of one pulse every 3.0 s in spring (for yearling Chinook salmon and juvenile steelhead) and 4.2 s in summer (for subyearling Chinook salmon). The nominal tag life was approximately 30 days in spring and 40 days in summer. Reliable, high resolution, 3-D tracking results for each individual fish were obtained using an advanced approximate maximum likelihood solver^39^.

### Analysis Methods

An approach was developed to evaluate the acclimation depth of seaward-migrating juvenile salmonids prior to dam passage^28^. For each fish, acclimation depth was defined as the depth at which this fish swam for the longest time within a defined area. The area was defined by setting horizontal distance limits (at least 25 m away from dam face) to exclude the period when fish were close to the powerhouse or spillway, and thus influenced by water velocities. The area also was restricted by the tracking accuracy observed during controlled field testing. Sub-meter tracking accuracy of individual tagged fish locations was achievable within up to 75 m of the horizontal distance to the face of the dam, while sub-meter accuracy was attained within 50 m downstream and upstream of the cabled hydrophone array at LFB. Before approaching the dam face (25–75 m from the dam face), the depth distributions of juvenile salmonids were estimated and compared according to their final passage routes.

Several large data sets were analyzed in this study: 1) Yearling Chinook salmon and juvenile steelhead from the 2012 Spring; 2) Subyearling Chinook salmon from the 2012 Summer; and 3) Subyearling Chinook salmon from the 2013 Summer. Each of the mid-reach vs. forebay comparisons includes one species (subyearling Chinook salmon), one mid-reach location (LFB), and two dams (LMN and LGS).

For each sample group, the acclimation depth was calculated for each individual fish and a set of acclimation depth values were recorded for the group. To conduct pairwise comparisons of the depth distribution between two different sample groups, statistical analyses were performed to investigate if the two samples followed the same distribution at a specific significance level^28^. For each sample group, a two-stage bootstrapping procedure was used to construct a 95% confidence interval (CI) for the observed cumulative distribution function from 1,000 bootstrapped simulations. A modified version of a Kolmogorov-Smirnov test of equal distributions was performed between two sample groups because acclimation depth values were measured with errors. Under the null hypothesis of equal distributions, the data from two alternative sample distributions were generated from a common distribution. As such, the distribution for the T-statistic can be empirically approximated by bootstrapping the pooled data from two alternative sample distributions. The detailed procedure was presented in Li *et al*.^28^. A P-value ≥ 0.05 was used as the criterion that the two sample groups were not significantly different. The most common depth was the depth which the majority (95%) of fish resided shallower than. It is the maximum residence depth for a certain fish group.

### Diel Period

Diel period was defined according to civil twilight times, which were used as the dividing points to categorize the fish into two sample groups—day and night. The start of morning civil twilight and end of evening civil twilight varied from April to August. Two separated dashed areas in Figs 4–6 were used to present the varied twilight periods for morning and evening. A fish was placed in the “day” group if it had more (>50%) day-time tracked points than night-time tracked points otherwise it was placed in the “night” group. Comparisons were performed to determine whether different fish species tended to occupy different depths in the water column during the day and night.

Data analyses were also performed by averaging median depth values during each hour of the 24-hour diel period in each area of concern. Median depth of a certain fish group for each hour was obtained by calculating the median value of all the fish in this group. Another metric investigated was if there was a preference of hours during a 24-hour diel period during which a higher percentage of passage for juvenile fish was observed. The number of salmonids passed through the different passage routes at the dams was counted for each hour of the day.

### Passage Probability

To answer the question “What was the probability (preference) of a fish being detected at a specific location in the forebay and ultimately passing through a certain passage route?” 3-D contour maps illustrating the passage probability were generated. The accuracy of the passage probability analysis will be restricted to the size of the data sets. The volume of the dam forebay was divided into 5 m x 5 m x 5 m cells, and the number of fish detected within a cell were counted over the season. Among the fish detected in each cell, the numbers of fish that passed different routes were collected to calculate the probabilities of different routes for each cell. The size of the data sets determines the base number of the counting for each cell. Larger data sets result in more accurate calculation of the probabilities for different routes in each cell, and decrease the occurrence of false high probabilities.

### Data Availability

All data generated and analyzed during this study are included in this published article (and its Supplementary Information files). More details are available from the corresponding author on reasonable request.

## Electronic supplementary material

Supplementary Information
