# Supplementary material for: Three-dimensional migration behavior of juvenile salmonids in reservoirs and near dams
Source: Sci Rep. 2018 Jan 17;8:956. doi: 10.1038/s41598-018-19208-1 (PMC5772619; doi:10.1038/s41598-018-19208-1)
Supplement: Supplementary file 1 — Supplementary Information [file 41598_2018_19208_MOESM1_ESM.pdf]

Supplementary Information

Three-dimensional migration behavior of juvenile salmonids in reservoirs and near dams

Xinya Li[1], Zhiqun D. Deng[1,*], Tao Fu[1], Richard S. Brown[1], Jayson J. Martinez[1], Geoffrey A. McMichael[2], Bradly A. Trumbo[3], Martin L. Ahmann[3], Jon F. Renholds[3], John R. Skalski[4], Richard L. Townsend[4]

[1]Pacific Northwest National Laboratory, P.O. Box 999, MSIN K9-33, Richland, WA 99332, USA

[2]Formerly at Pacific Northwest National Laboratory, Currently with Mainstem Fish Research

[3]U.S. Army Corps of Engineers, Walla Walla District, 201 N Third Ave, Walla Walla, WA 99362, USA

[4]School of Aquatic and Fishery Sciences, University of Washington, 1325 Fourth Avenue, Suite 1820, Seattle, WA 98101, USA

*Corresponding Author

Telephone: +1 509 372 6120; Fax: +1 509 372 6089; Email address: zhiqun.deng@pnnl.gov

Table S1: Median depth at which subyearling Chinook salmon were detected at LFB during 2013. The compared sample groups were selected based on their routes at the upstream detection site (LGS) or the downstream detection site (LMN). 95% CI are listed in parentheses. The depth at which 95% of the fish were found at or were shallower than is also shown.

			Sample Group A			Sample Group B		
Sample Group A	Sample Group B	P	Sample Size	Most Common Depth (95%) (m)	Median Depth (m)	Sample Size	Most Common Depth (95%) (m)	Median Depth (m)
SP at LGS	PH at LGS	0.28	1594	**12.1** (11.7, 12.9)	**5.0** (4.7, 5.4)	492	11.6 (11.1, 12.3)	**5.1** (4.5, 5.5)
RS at LGS	SW at LGS	0.89	217	**13.2** (11.8, 14.5)	**5.1** (4.3, 6.2)	1377	12.0 (11.5, 12.8)	**5.0** (4.7, 5.4)
Turbine at LGS	JBS at LGS	0.90	104	**11.8** (10.3, 13.8)	**5.3** (3.8, 6.6)	388	11.4 (11.0, 12.3)	**5.1** (4.5, 5.6)
SP at LMN	PH at LMN	0.21	3075	**12.4** (12.1, 12.8)	**5.0** (4.8, 5.2)	345	12.7 (12.0, 14.4)	**5.5** (4.8, 6.1)
RS at LMN	SW at LMN	0.76	741	**12.0** (11.4, 12.8)	**5.0** (4.5, 5.3)	2334	12.5 (12.2, 13.1)	**5.0** (4.8, 5.3)
Turbine at LMN	JBS at LMN	0.14	140	**13.1** (12.1, 16.9)	**6.0** (4.8, 7.0)	205	12.4 (11.2, 14.1)	**5.2** (4.4, 6.0)

SP at LGS = Fish at LFB with spillway passage at upstream LGS; PH at LGS = Fish at LFB with powerhouse passage at upstream LGS
RS at LGS = Fish at LFB with regular-spillway passage at upstream LGS; SW at LGS = Fish at LFB with spillway-weir passage at upstream LGS
Turbine at LGS = Fish at LFB with turbine passage at upstream LGS; JBS at LGS = Fish at LFB with JBS passage at upstream LGS
SP at LMN = Fish at LFB with spillway passage at downstream LMN; PH at LMN = Fish at LFB with powerhouse passage at downstream LMN
RS at LMN = Fish at LFB with regular-spillway passage at downstream LMN; SW at LMN = Fish at LFB with spillway-weir passage at downstream LMN
Turbine at LMN = Fish at LFB with turbine passage at downstream LMN; JBS at LMN = Fish at LFB with JBS passage at downstream LMN

Table S2: Median depth at which three types of juvenile salmonids were detected within the forebay of LGS and LMN during 2012 and 2013 passed through the regular spillway or spillway weir. 95% CI are listed in parentheses. The depth at which 95% of the fish were found at or were shallower than is also shown. All of the comparisons of depth distribution between regular-spillway-passed and spillway-weir-passed were significantly different (P<0.001).

			Regular Spillway Passage			Spillway Weir Passage		
Year	Species	Location	Sample Size	Most Common Depth (95%) (m)	Median Depth (m)	Sample Size	Most Common Depth (95%) (m)	Median Depth (m)
2012	CH1	LGS	301	**11.8** (9.9, 13.6)	**2.9** (2.1, 3.7)	746	**7.0** (6.5, 7.9)	**1.0** (0.9, 1.2)
2012	ST	LGS	242	**17.9** (14.8, 20.1)	**3.4** (2.5, 4.8)	675	**8.9** (8.2, 10.0)	**0.9** (0.9, 1.0)
2012	CH0	LGS	602	**15.7** (14.1, 18.3)	**3.9** (3.5, 4.5)	1264	**10.8** (10.4, 11.6)	**3.8** (3.5, 4.1)
2012	CH1	LMN	623	**8.8** (7.9, 9.5)	**1.1** (1.1, 1.4)	2433	**5.7** (5.5, 6.1)	**1.1** (1.1, 1.2)
2012	ST	LMN	497	**16.9** (14.6, 20.4)	**4.8** (4.1, 5.5)	2106	**8.9** (8.5, 9.4)	**1.1** (1.2, 1.2)
2012	CH0	LMN	1535	**14.0** (12.9, 14.8)	**4.0** (3.9, 4.2)	3590	**10.3** (10.1, 11.0)	**3.9** (3.8, 4.0)
2013	CH0	LGS	246	**14.6** (13.3, 15.6)	**8.1** (6.9, 8.5)	1570	**12.9** (12.7, 13.3)	**6.1** (5.9, 6.6)
2013	CH0	LMN	1163	**15.6** (15.1, 16.4)	**7.0** (6.6, 7.4)	3582	**13.6** (13.3, 13.9)	**5.7** (5.6, 6.0)

CH0 = subyearling Chinook salmon; CH1 = yearling Chinook salmon; ST = steelhead

Table S3: Median depth at which three types of juvenile salmonids were detected within the forebay of LGS and LMN during 2012 and 2013 passed regular spillway and JBS. 95% CI are listed in parentheses. The depth at which 95% of the fish were found at or were shallower than is also shown.

Year	Species	Location	P	Regular Spillway Passage			JBS Passage		
				Sample Size	Most Common Depth (95%) (m)	Median Depth (m)	Sample Size	Most Common Depth (95%) (m)	Median Depth (m)
2012	CH1	LGS	<0.001	301	**11.8** (9.9, 13.6)	**2.9** (2.1, 3.7)	545	**13.3** (11.8, 15.1)	**3.0** (2.6, 3.7)
2012	ST	LGS	0.01	242	**17.9** (14.8, 20.1)	**3.4** (2.5, 4.8)	730	**15.2** (13.7, 17.0)	**4.9** (4.5, 5.5)
2012	CH0	LGS	0.01	602	**15.7** (14.1, 18.3)	**3.9** (3.5, 4.5)	632	**15.9** (14.8, 18.2)	**3.9** (3.4, 4.8)
2012	CH1	LMN	<0.001	623	**8.8** (7.9, 9.5)	**1.1** (1.1, 1.4)	631	**11.0** (10.2, 11.9)	**2.8** (2.2, 3.1)
2012	ST	LMN	<0.001	497	**16.9** (14.6, 20.4)	**4.8** (4.1, 5.5)	1220	**16.1** (15.2, 18.1)	**6.1** (5.8, 6.5)
2012	CH0	LMN	<0.001	1535	**14.0** (12.9, 14.8)	**4.0** (3.9, 4.2)	549	**16.8** (15.9, 19.4)	**4.7** (4.3, 5.6)
2013	CH0	LGS	<0.001	246	**14.6** (13.3, 15.6)	**8.1** (6.9, 8.5)	470	**19.9** (17.9, 22.0)	**9.5** (8.8, 10.4)
2013	CH0	LMN	<0.001	1163	**15.6** (15.1, 16.4)	**7.0** (6.6, 7.4)	319	**21.2** (18.1, 23.9)	**8.9** (7.7, 9.9)

CH0 = subyearling Chinook salmon; CH1 = yearling Chinook salmon; ST = steelhead

Table S4: Number of acoustic tagged subyearling Chinook salmon that passed LGS through different routes in 2013 for each hour of the day. Daytime hours = 5-19 and nighttime hours = 20-4.

Hour	SW	RS	Turbine	JBS	SP (SW + RS)	PH (Turbine + JBS)	Total (SP+PH)
0	31	8	29	38	39	67	106
1	36	6	17	42	42	59	101
2	29	10	10	34	39	44	83
3	38	6	13	30	44	43	87
4	43	4	0	10	47	10	57
5	44	6	0	4	50	4	54
6	55	17	1	11	72	12	84
7	56	17	3	8	73	11	84
8	62	19	0	18	81	18	99
9	94	25	4	15	119	19	138
10	111	35	2	26	146	28	174
11	120	28	2	27	148	29	177
12	100	18	3	28	118	31	149
13	152	21	1	18	173	19	192
14	129	18	0	22	147	22	169
15	128	15	0	9	143	9	152
16	120	19	1	10	139	11	150
17	92	12	0	3	104	3	107
18	75	8	0	1	83	1	84
19	54	3	0	2	57	2	59
20	42	1	2	3	43	5	48
21	19	2	4	10	21	14	35
22	19	9	14	49	28	63	91
23	29	9	23	54	38	**77**	115
	1678	316	129	472	1994	601	2595

RS = Fish with regular-spillway passage; SW = Fish with spillway-weir passage

Turbine = Fish with turbine passage; JBS = Fish with JBS passage

SP = Fish with spillway passage ; PH = Fish with powerhouse passage
